# Supplementary material for: Transcriptional regulatory networks underlying gene expression changes in Huntington's disease
Source: Mol Syst Biol. 2018 Mar 26;14(3):e7435. doi: 10.15252/msb.20167435 (PMC5868199; doi:10.15252/msb.20167435)
Supplement: Supplementary file 1 — Appendix [file MSB-14-e7435-s001.pdf]

Appendix Information

Appendix Figure S1. Comparison of TF binding site predictions to ChIP-seq data. For each of 52 TFs, we compared the sets of genes adjacent to predicted TF binding sites in our model to the sets of genes adjacent to observed binding sites from ChIP-seq studies. **a.** $-\log_{10}$(p-values) for overlap between modeled vs. observed gene sets (Fisher's exact test). **b.** Distribution of recall (sensitivity) and precision (positive predictive accuracy) of the TFBS model for identifying the target genes of each TF identified by ChIP-seq.

Appendix Figure S2. Association between TRN prediction accuracy and expression level. Each point on the scatterplot represents the mean expression level of a gene in the striatum (x-axis; fragments per kilobase million, FPKM) and the prediction accuracy for that gene in the transcriptional regulatory network model (r^2, predicted vs. observed expression across all samples).

Appendix Figure S3. TFs with >1,000 predicted target genes. Bars indicate the number of predicted target genes for each of the 15 TFs with >1,000 predicted target genes in the TRN model for the mouse striatum.

Appendix Figure S4. Enrichments of TF modules within each striatal cell type. Enrichments of the predicted target genes of each TF for genes expressed specifically in one of seven major cell types in the mouse striatum. The top 20 TF modules are shown for each cell type, ranked by the –log10(p-value) for the strength of enrichment in a one-sided Fisher's exact test.

Appendix Figure S5. Core regulator TFs are differentially expressed in the striatum of HD CAG knock-in mice. z-scores indicate significance and direction of expression changes in each condition, relative to age-matched $Htt^{Q20/+}$ mice.

Appendix Figure S6. Reconstruction of a TRN model of the human striatum. a. Training (black) and test set (blue) prediction accuracy for genes in the human striatum TRN model. b. Distribution for the number of predicted regulators per target gene. c. Distribution for the number of predicted target genes per TF. d. Enrichment of down-regulated core regulator TFs identified in mouse striatum for down-regulated genes in HD cases vs. controls. e. Enrichment of up-regulated core regulator TFs identified in mouse striatum for up-regulated genes in HD cases vs. controls.

Appendix Figure S7. Enrichments of target genes of striatal core regulator TFs for Htt CAG-length dependent gene expression changes in cortex, hippocampus, cerebellum, and liver. Heatmap color indicates the –log10(p-value) for the enrichment of each TF's predicted target genes in each tissue (Fisher's exact test). In each tissue, we tested for enrichment separately in each age x Htt allele condition (three ages: 2-month-old, 6-month-old, 10-month-old; five genotypes: Q80, Q92, Q111, Q140, Q175). In addition, enrichments were calculated separately for down-regulated and for up-regulated genes (edgeR, $p < 0.01$). The color on the heatmap corresponds to the lowest enrichment p-value for each TF within each tissue. The 13 TFs whose target genes were consistently enriched for gene expression changes across four

independent striatal datasets are indicated in bold with asterisks. STR, striatum; CTX, cortex; HIP, hippocampus; CB, cerebellum.

Appendix Figure S8. Effects of age- and *Htt* genotype on abundance of SMAD3 and phospo-Ser423/425-SMAD3 protein. a. Representative western blots of striatal tissue from 4- and 11-month-old $Htt^{Q111/+}$ and wildtype mice. Actin levels and two mice per genotype and age are shown for illustrative purposes. b. Quantification of phospho-SMAD3 signal (ANOVA, Genotype: $F_{(1,16)} = 2.713$, $p = 0.119$; Age: $F_{(1,16)} = 6.509$, $p = 0.021$; Interaction: $F_{(1,16)} = 0.007$, $p = 0.934$). c. Quantification of total SMAD3 signal (ANOVA, Genotype: $F_{(1,16)} = 0.487$, $p = 0.495$; Age: $F_{(1,16)} = 0.506$, $p = 0.487$; Interaction: $F_{(1,16)} = 1.085$, $p = 0.313$). d. phospho-SMAD3 to total SMAD3 ratio (ANOVA, Genotype: $F_{(1,16)} = 3.714$, $p = 0.072$; Age: $F_{(1,16)} = 4.590$, $p = 0.048$; Interaction: $F_{(1,16)} = 0.304$, $p = 0.589$). Box plots: box represents first and third quartiles, whiskers represent data within 1.5 times the interquartile range (IQR), and points represent any data beyond 1.5 times the IQR.

Appendix Table S1. GO enrichments of top 837 SMAD3 target genes.

Appendix Figure S1

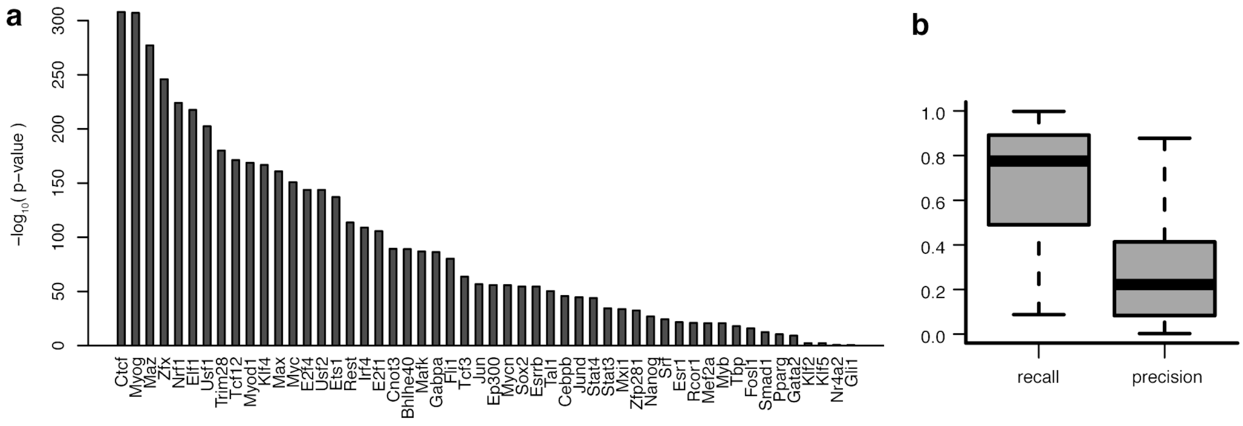

Appendix Figure S2

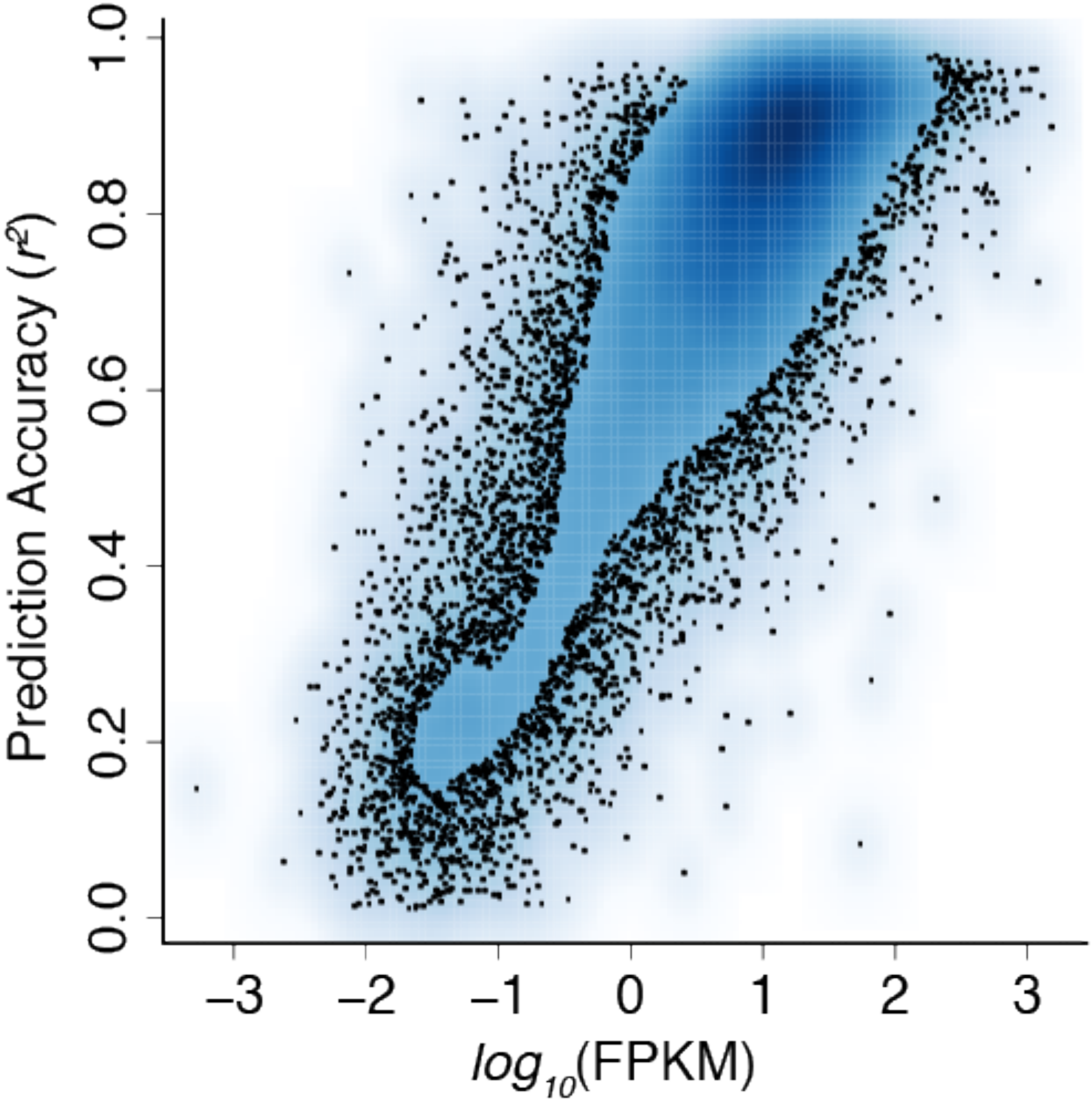

Appendix Figure S3

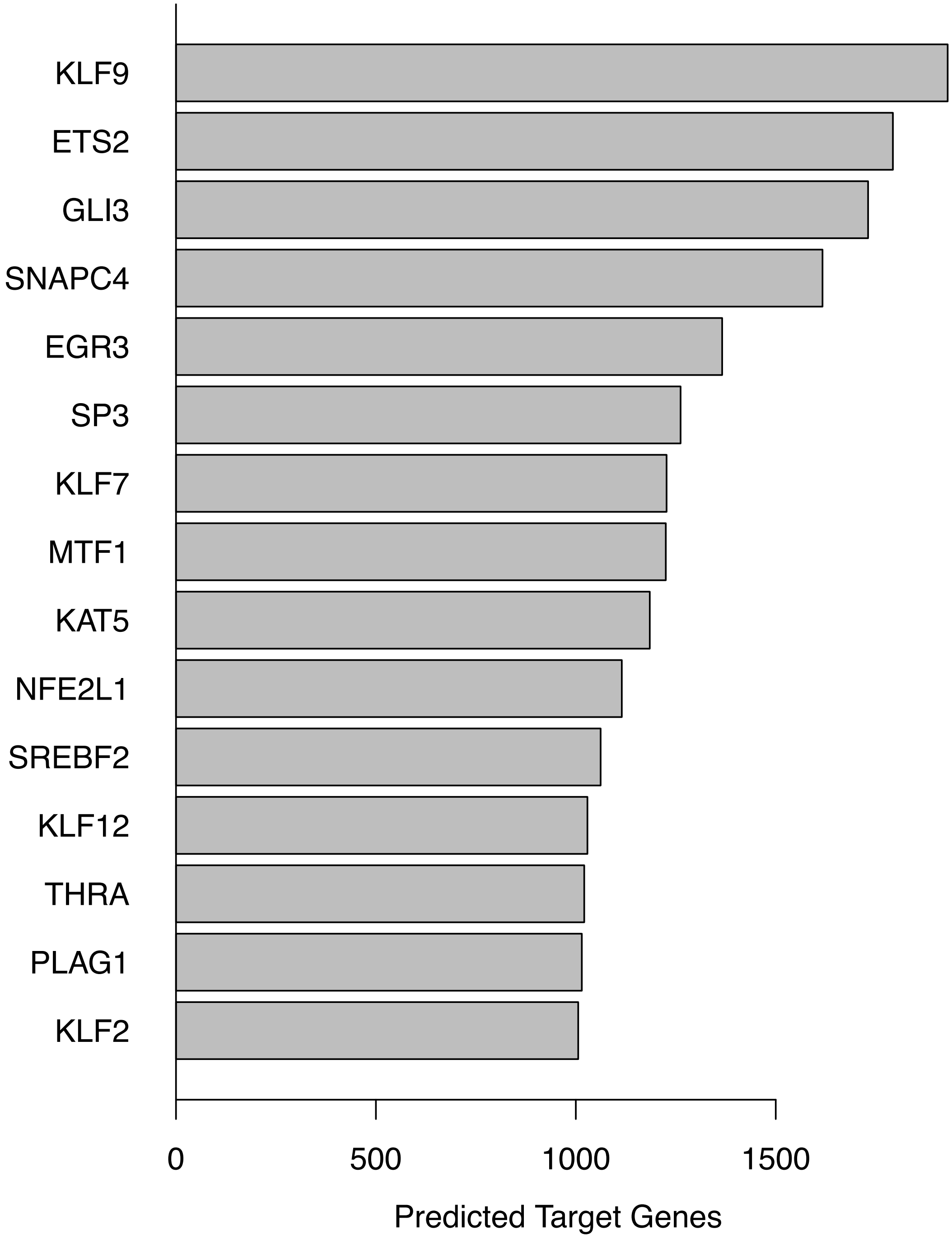

Appendix Figure S4

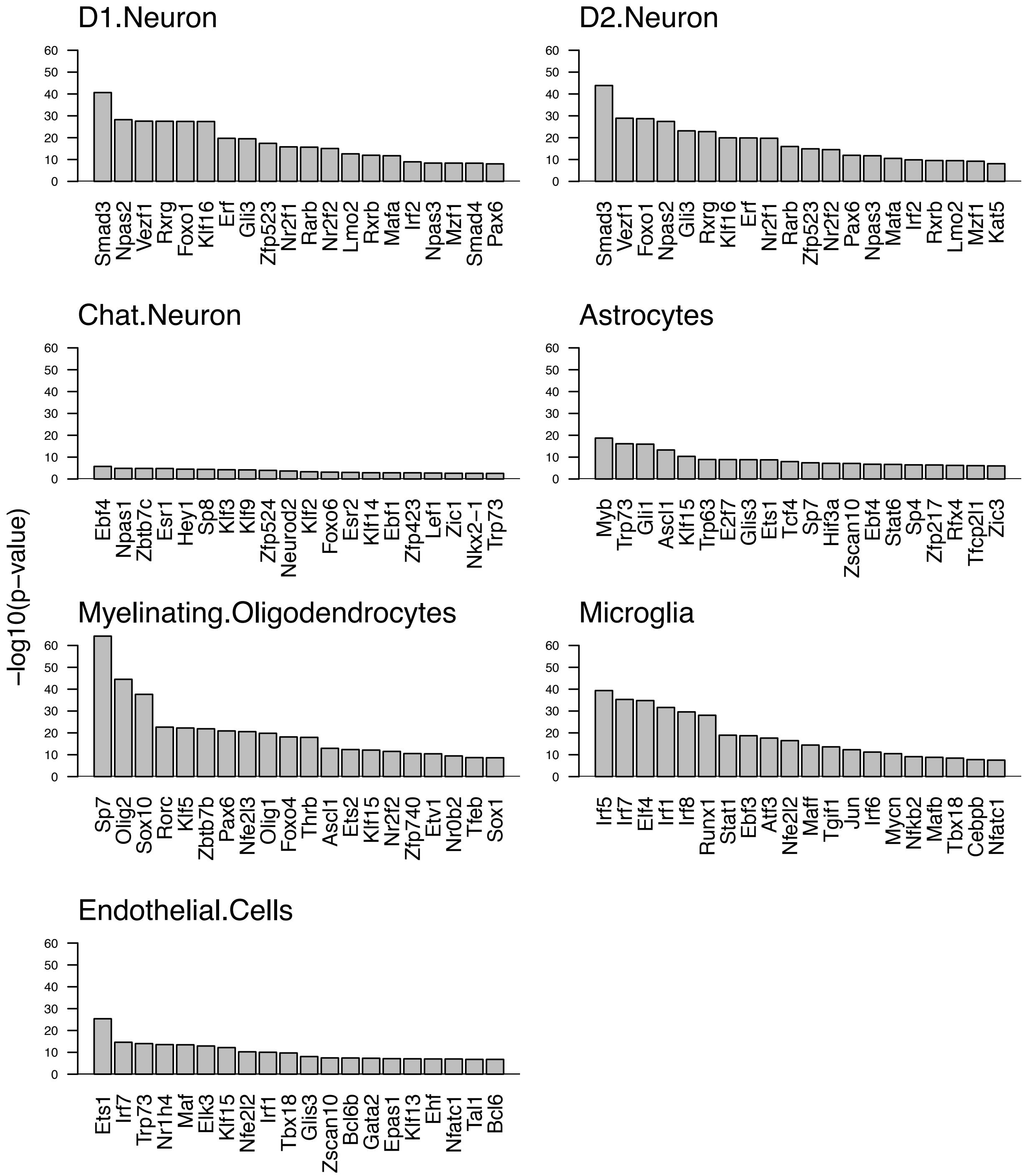

Appendix Figure S5

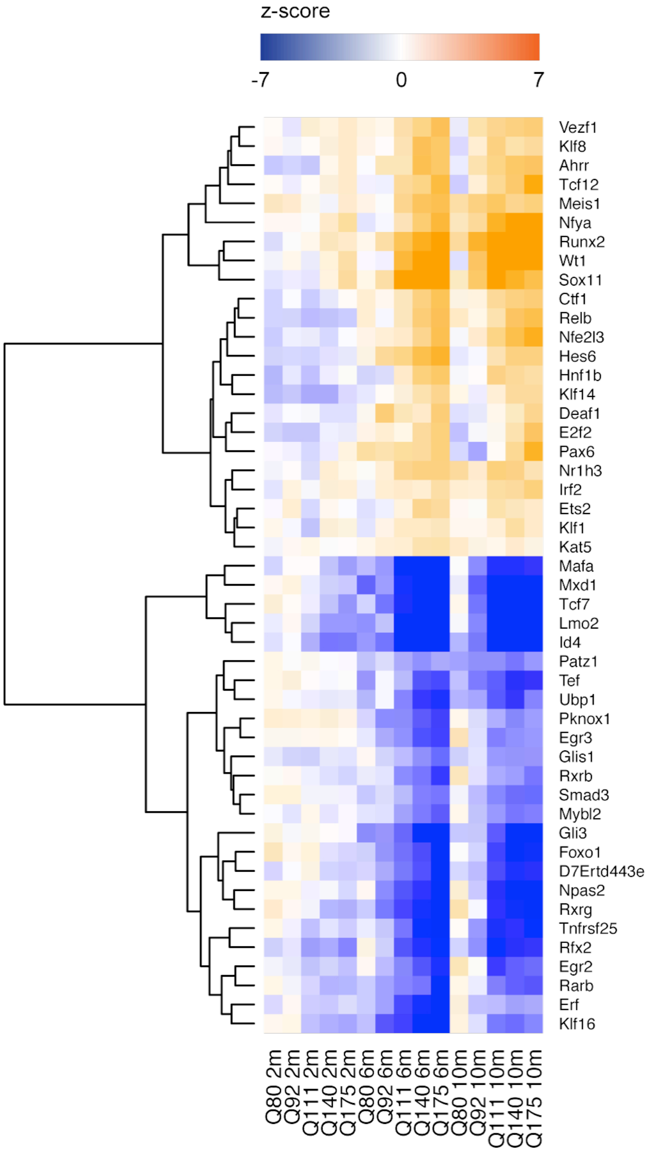

Appendix Figure S6

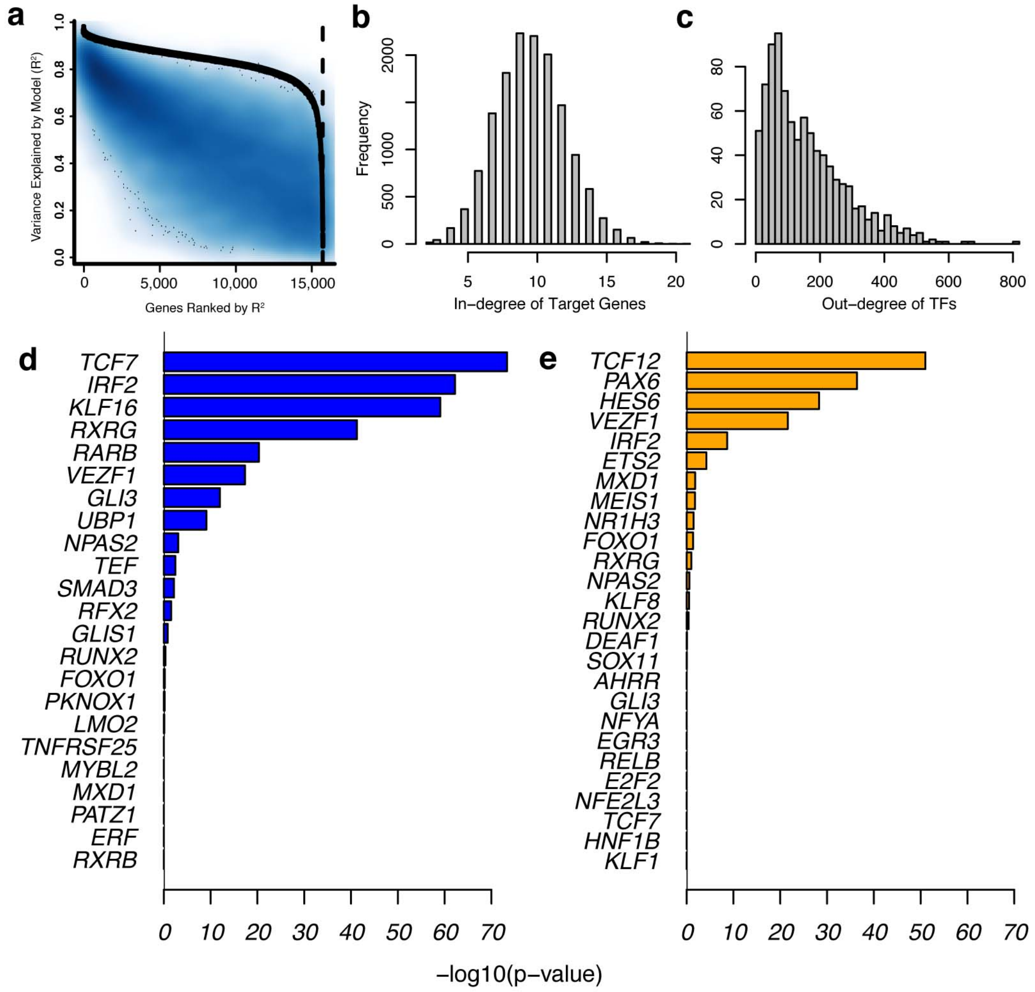

Appendix Figure S7

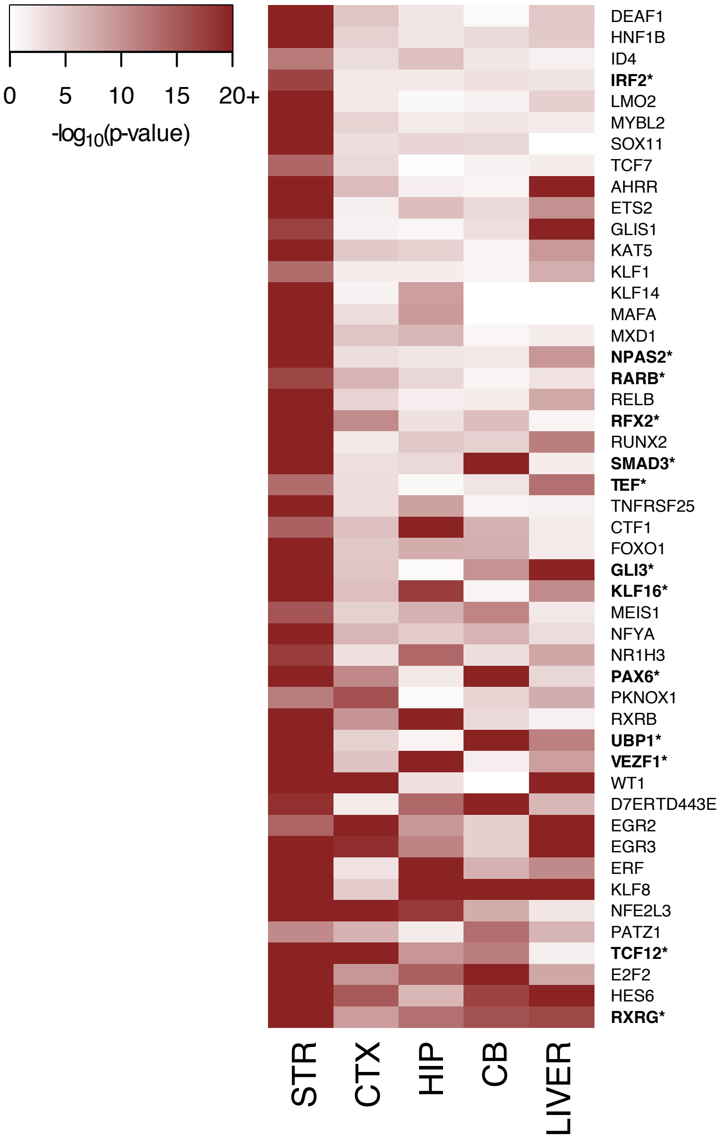

Appendix Figure S8

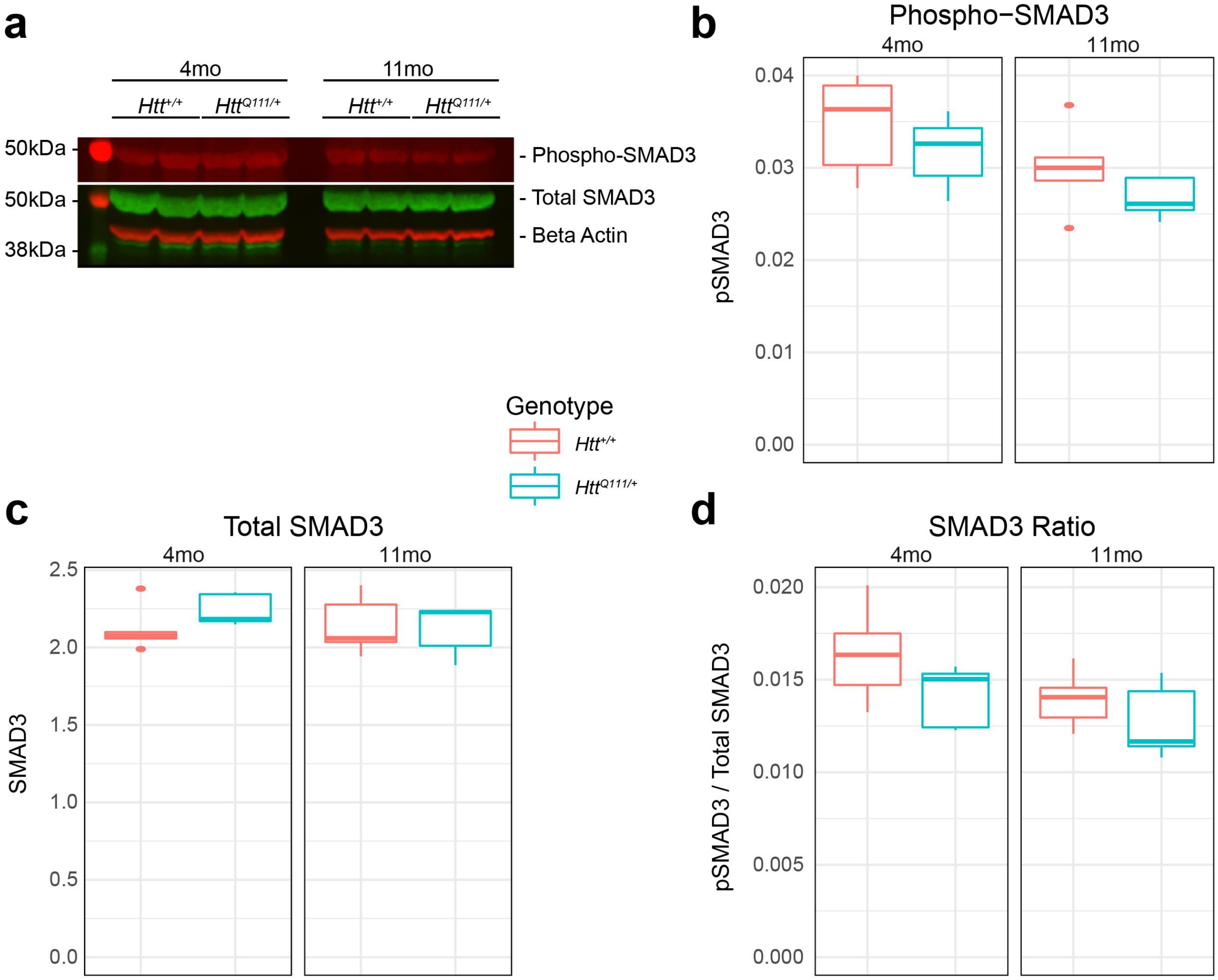

Appendix Table S1 GO enrichments of top 837 SMAD3 target genes.

Term	Set Size	SMAD3 Targets	Odds Ratio	P-Value	FDR
Actin Filament-Based Process	442	41	3.6	4.2E-11	1.9E-07
mRNA Processing	344	32	3.7	4.2E-09	9.6E-06
Acting Binding	332	30	3.6	2.1E-08	2.4E-05
Neuromuscular Process Controlling Balance	59	12	8.5	1.2E-07	9.4E-05
Histone Modification	293	26	3.6	1.7E-07	1.1E-04
Brain Development	432	32	2.8	1.3E-06	4.9E-04
Chromatin Binding	387	29	2.8	2.7E-06	9.3E-04
Actin Filament-Based Movement	65	11	6.8	2.8E-06	9.3E-04
Regulation of Cell Projection Organization	380	28	2.8	4.5E-06	1.3E-03
Lamellipodium	114	14	4.8	5.5E-06	1.4E-03
Protein Serine/Threonine Kinase Activity	409	29	2.6	9.9E-06	2.3E-03
Protein Deacetylation	49	9	7.5	1.1E-05	2.4E-03
Centrosome	350	25	2.8	1.4E-05	2.6E-03
Purine Ribonucleotide Catabolic Process	382	27	2.7	1.6E-05	3.0E-03
Kinase Binding	199	18	3.4	2.3E-05	4.0E-03
Phosphoric Ester Hydrolase Activity	374	26	2.6	2.5E-05	4.0E-03
Neuronal Cell Body	411	28	2.5	2.5E-05	4.0E-03
Protein Kinase Binding	369	26	2.6	2.8E-05	4.2E-03
Cellular Protein Catabolic Process	415	28	2.5	3.0E-05	4.2E-03
Transcriptional Repressor Complex	70	10	5.6	3.7E-05	4.4E-03
Respiratory System Development	138	14	3.8	5.5E-05	5.3E-03
Kinesin Binding	29	6	9.5	1.1E-04	9.3E-03
Endocytosis	334	23	2.6	1.1E-04	9.3E-03
Negative Regulation of ERBB Signaling Pathway	10	4	22.1	1.4E-04	9.7E-03
