# Supplementary material for: Transcriptional regulatory networks underlying gene expression changes in Huntington's disease
Source: Mol Syst Biol. 2018 Mar 26;14(3):e7435. doi: 10.15252/msb.20167435 (PMC5868199; doi:10.15252/msb.20167435)
Supplement: Supplementary file 2 — Dataset EV1 [file MSB-14-e7435-s002.zip › README_for_DATASET_EV1.docx]

README for DATASET EV1

Transcriptional regulatory network model for the mouse striatum.

Each row corresponds to a single TF-gene interaction predicted by our model.

Column 1: transcription factor

Column 2: predicted target gene

Column 3: beta coefficient from the LASSO regression model, indicating the direction and strength of the effect of the transcription factor on the predicted target gene.
