# Supplementary material for: Transcriptional regulatory networks underlying gene expression changes in Huntington's disease
Source: Mol Syst Biol. 2018 Mar 26;14(3):e7435. doi: 10.15252/msb.20167435 (PMC5868199; doi:10.15252/msb.20167435)
Supplement: Supplementary file 3 — Dataset EV2 [file MSB-14-e7435-s003.zip › README_for_DATASET_EV2.docx]

README for DATASET EV2

**Enrichments of TF-target gene modules for striatal gene expression changes in mouse models of Huntington's disease and in Huntington's disease cases vs. controls.**Enrichments of each TF's predicted target genes for down- and up-regulated genes in RNA-seq, microarray, and proteomics datasets from mouse striatum, and in microarray gene expression profiles from human striatum.

This dataset contains the following tabs and columns:

Mouse meta-analysis

- Module: Sets of genes regulated by each transcription factor in the mouse striatum TRN model
- GSE65776: Meta-analytic p-value for enrichment of each TF’s predicted target genes among differentially expressed genes across the 15 conditions in Langfelder et al. RNA-seq of mouse striatum. Enrichments for down- and up-regulated genes were tested separately.
- Microarray Datasets: Meta-analytic p-value for enrichment of each TF’s predicted target genes among differentially expressed genes across four independent microarray gene expression profiling experiments using striatal tissue from HD mouse models.
- Proteomics Datasets: Enrichment of each TF’s predicted target genes among differentially expressed genes in proteomics data from Langfelder et al. 2016.

Human-mouse meta-analysis: This tab shows the same data as the Mouse Meta-Analysis tab, plus additional columns corresponding to data from human striatum and human neural stem cells. Note: Only those TFs with one-to-one mouse-human orthologs are shown in this tab.

The remaining tabs show contingency tables, p-values, and q-values for enrichments of each TF’s predicted target genes among differentially expressed genes in individual datasets.
