# Supplementary material for: Transcriptional regulatory networks underlying gene expression changes in Huntington's disease
Source: Mol Syst Biol. 2018 Mar 26;14(3):e7435. doi: 10.15252/msb.20167435 (PMC5868199; doi:10.15252/msb.20167435)
Supplement: Supplementary file 4 — Dataset EV3 [file MSB-14-e7435-s004.zip › README_for_DATASET_EV3.docx]

README for DATASET EV3

Enrichments of each TF's predicted target genes among Huntington's disease related gene expression changes in striatum, cortex, hippocampus, cerebellum, and liver, using data from Langfelder et al. 2016.

Column 1: transcription factor

Columns 2-11: p-values for the strength of association between each transcription factor’s predicted target genes with up- or down-regulated genes in this tissue. Str: striatum; ctx: cortex; hip: hippocampus; cb: cerebellum.
